# Association Between Transvaginal Ultrasound Measurement of Endometrial Thickness With Serum Estrogen Level in Post-menopausal Women in a Tertiary Care Centre

**DOI:** 10.7759/cureus.94154

**Published:** 2025-10-08

**Authors:** Pratikshya Priyadarshini, TG Revathy, Preethika A

**Affiliations:** 1 Obstetrics and Gynaecology, Sree Balaji Medical College and Hospital, Chennai, IND

**Keywords:** endometrium, estrogen and cancer, gynaecological evaluation, post-menopause, transvaginal ultrasound scan

## Abstract

Background

Estrogen plays a vital role in endometrial physiology, and its deficiency in postmenopausal women leads to endometrial atrophy. However, residual or elevated estrogen levels - endogenous or exogenous - can stimulate endometrial thickening, which may signal an underlying pathology. Transvaginal ultrasound (TVUS) offers a non-invasive, practical method to measure endometrial thickness (ET), potentially serving as a surrogate marker for systemic estrogen exposure. This prospective observational study aims to correlate serum estradiol levels with endometrial thickness in postmenopausal women and to analyze the association between endometrial thickness and known estrogen-related risk factors.

Methods

A total of 100 postmenopausal women attending the gynaecology outpatient department at Sree Balaji Medical College and Hospital, Chennai, over a span of two years, from 2023 to 2025, were evaluated using TVUS for endometrial thickness and serum assay for estradiol levels. Baseline characteristics and comorbid conditions such as obesity, diabetes mellitus, hypertension, and parity status were recorded. Statistical analysis was used to assess correlations and significance.

Results

The mean endometrial thickness was 6.1±2.3 mm, and the mean serum estradiol level was 24.8±9.6 pg/mL. A moderate to strong positive correlation (r=0.78, p<0.001) was observed between serum estradiol levels and endometrial thickness. Endometrial thickness >4 mm was significantly associated with obesity (p=0.003), diabetes mellitus (p=0.005), and nulliparity (p = 0.008), but not with hypertension (p=0.082).

Conclusion

Serum estradiol levels may act as a surrogate biochemical marker for increased endometrial thickness in postmenopausal women. While elevated estrogenic activity can contribute to endometrial proliferation, increased endometrial thickness should not be interpreted in isolation as a direct indicator of high estrogen levels. Integrating serum hormone evaluation with TVUS findings and clinical risk profiling may enhance the early identification of women predisposed to estrogen-related endometrial pathology, particularly in postmenopausal care settings.

## Introduction

Menopause, marking the permanent cessation of ovarian follicular function and menstruation, is a universal and significant physiological transition in a woman's life. This event is characterized by a profound decline in the production of sex steroids, particularly estradiol, leading to the end of reproductive capability and initiating a new endocrine milieu [1]. The hypoestrogenic state that defines postmenopause is not merely a gynecological endpoint but a systemic event associated with a wide spectrum of clinical sequelae. These include well-documented vasomotor symptoms (hot flashes), genitourinary syndrome of menopause (vulvovaginal atrophy, dyspareunia), osteoporosis, and alterations in cardiovascular and cognitive health [2,3]. The management of these symptoms and the long-term health risks associated with estrogen deficiency remain a central focus of clinical gynecology.

Among the most sensitive tissues to estrogenic stimulation is the endometrium, the lining of the uterus. Throughout a woman's reproductive life, the endometrium undergoes cyclical proliferation, secretion, and shedding in response to the fluctuating levels of estrogen and progesterone. The postmenopausal state, characterized by the absence of this cyclical hormonal stimulation, typically results in endometrial atrophy. In a healthy postmenopausal woman not on hormone therapy, the endometrium is expected to be thin, homogeneous, and quiescent [4]. This atrophic state is a direct consequence of low circulating estrogen levels and is considered a physiological norm.

However, the postmenopausal endometrium can still respond to estrogenic stimuli. In certain pathological contexts, such as unopposed estrogen production from ovarian stromal hyperplasia, peripheral aromatization of androgens in adipose tissue, or the presence of estrogen-secreting tumors, the endometrium may undergo proliferation [5]. Furthermore, the widespread use of exogenous estrogen, either as menopausal hormone therapy (MHT) or via selective estrogen receptor modulators (SERMs) like tamoxifen, can lead to endometrial thickening. This responsiveness makes the endometrium a potential biological indicator, or biomarker, of systemic estrogenic activity [6]. The critical clinical concern is that prolonged, unopposed estrogen exposure significantly elevates the risk of endometrial hyperplasia, a precursor lesion that can progress to endometrial carcinoma, the most common gynecologic malignancy in developed nations [7].

The accurate and non-invasive assessment of the postmenopausal endometrium is, therefore, of paramount importance. Transvaginal ultrasonography (TVUS) has emerged as the cornerstone imaging modality for this purpose. Its proximity to the pelvis provides high-resolution images of the uterus, allowing for precise measurement of endometrial thickness (ET). The sonographic ET is measured as the maximal anterior-posterior dimension of the endometrial echo complex on a sagittal view [8]. Due to its sensitivity, reproducibility, and patient acceptability, TVUS has become an indispensable tool in gynecological practice. A thin, distinct endometrial stripe (typically defined as ≤4 mm) is strongly associated with endometrial atrophy and is highly effective in ruling out significant pathology, with a negative predictive value for endometrial cancer exceeding 99% [9,10]. Consequently, a thin ET on TVUS is often reassuring and can prevent the need for more invasive diagnostic procedures. 

Conversely, an abnormally thickened endometrium in a postmenopausal woman is a common clinical dilemma that warrants further investigation. It serves as a non-invasive surrogate marker suggesting exposure to elevated levels of estrogen, whether endogenous or exogenous. While not diagnostic of pathology itself, increased ET triggers a differential diagnosis that includes benign conditions like polyps or cystic atrophy, but also necessitates the exclusion of hyperplasia and carcinoma, typically through histological evaluation via endometrial biopsy [8]. The correlation between ET and serum estrogen levels, however, is not always linear or straightforward. In essence, vitamin D may act as a modulator of estrogenic activity in endometrial tissue. Adequate vitamin D levels support balanced estrogen signaling, potentially preventing excessive endometrial proliferation. Conversely, vitamin D deficiency may contribute to endometrial thickening or hyperplastic risk, particularly in postmenopausal women with obesity or metabolic comorbidities. Factors such as individual endometrial sensitivity, duration of exposure, body mass index (BMI) - which influences endogenous estrogen production via aromatization - and the presence of local intrauterine pathologies can all modulate this relationship [11,12].

Despite the established clinical utility of TVUS, a clear and quantitative understanding of the correlation between directly measured serum estradiol levels and sonographic ET in a diverse postmenopausal population is still an area of active investigation. Many studies have focused on ET as a predictor of pathology, but fewer have prospectively examined its strength as a continuous biomarker for circulating estrogenic activity, while also controlling for confounding factors like BMI and parity.

The primary aim of this prospective observational study is to rigorously evaluate the relationship between serum estradiol levels and ET as measured by TVUS in a cohort of postmenopausal women. A secondary aim is to analyze the association between ET and other factors linked to cumulative estrogen exposure, such as BMI, age at menopause, and parity. By elucidating these relationships, this research seeks to enhance the diagnostic interpretation of TVUS findings, refine screening protocols for endometrial pathologies, and contribute to a more nuanced, non-invasive method for assessing estrogenic activity in the postmenopausal population. This could ultimately lead to improved, personalized risk stratification and clinical management for women during this pivotal life stage.

## Materials and methods

Study setting and place

The present observational study was conducted in the Department of Obstetrics and Gynaecology at Sree Balaji Medical College and Hospital, Chromepet, Chennai. The institution caters to a large population of women from diverse socioeconomic and demographic backgrounds, offering a robust base for clinical and research activities. The study was carried out among postmenopausal women attending the outpatient department (OPD) of Obstetrics and Gynaecology, thereby ensuring the inclusion of participants from a representative clinical pool.

Study design and duration

This study adopted an institutional, prospective observational design to investigate endometrial pathology in postmenopausal women. The research was conducted over a span of two years, from 2023 to 2025. This duration provided an adequate time frame for participant recruitment, data collection, and comprehensive evaluation using transvaginal ultrasonography (TVS).

Participant selection

Women attending the OPD who had attained natural menopause were approached for participation. In this study, natural menopause was defined as the spontaneous cessation of menstruation for at least 12 consecutive months in women aged 45 years or older, not attributable to surgery, chemotherapy, or other medical causes. In cases where menstrual history was uncertain, biochemical confirmation was obtained through serum follicle-stimulating hormone (FSH) levels exceeding 40 IU/L along with low serum estradiol concentrations (<30 pg/mL) on two separate assessments taken four weeks apart, thereby confirming the postmenopausal status. Following an explanation of the study objectives and procedures, written informed consent was obtained from each participant. Only those individuals who provided consent and fulfilled the inclusion criteria were enrolled in the study.

Inclusion and exclusion criteria

Participants were included if they were postmenopausal women who had attained natural menopause and were not receiving hormone replacement therapy (HRT). Women below the age of 45 years, those currently on tamoxifen therapy, individuals previously diagnosed with malignant endometrial hyperplasia with atypia or adenocarcinoma, and those unwilling to provide consent were excluded from the study. These exclusion criteria were established to minimize the confounding factors and ensure a homogenous study cohort focusing on naturally postmenopausal women. Women who had undergone prior TVUS or pelvic imaging for endometrial assessment within the preceding six months were excluded. Post-hysterectomy cases were also excluded from the study.

Sample size determination

The sample size was calculated using the formula based on evidence from a previous study. The expected proportion of endometrial pathology in postmenopausal women was considered to be 7% [13]. An absolute error of 5% was allowed and at 80% power, a total of 100 participants were recruited for this study.

Study procedure

Preparation for Transvaginal Sonography

All the enrolled participants underwent TVS using a 7.5 MHz transvaginal sector probe (GE Voluson E8, GE Healthcare, Chicago, IL, USA) equipped with phased array and end-firing capabilities. Prior to the procedure, participants were instructed to empty their bladders completely to enhance the clarity of the images obtained. The probe was prepared under aseptic conditions, covered with a sterile sheath or condom, and coated with acoustic coupling gel to maximize transmission quality.

Patient Positioning and Probe Introduction

During the procedure, all women were positioned comfortably in the supine posture, and the transducer was carefully introduced into the posterior vaginal fornix to achieve optimal visualization of pelvic structures while minimizing discomfort. The uterus was initially evaluated in both long axis and coronal views, with the sagittal plane serving as the primary orientation. Scanning was performed systematically from the fundus down to the internal os to assess uterine size, morphology, and overall regularity. Uterine measurements, including length, anteroposterior, and transverse dimensions, were obtained, and endometrial volume was subsequently calculated. Endometrial thickness was measured in the anteroposterior dimension, extending from one basalis layer to the contralateral basalis along the central longitudinal axis. To ensure accuracy, oblique or semicoronal views were strictly avoided as they could result in exaggerated measurements. Careful evaluation of the endometrial architecture was undertaken to assess homogeneity, echotexture, and the integrity of the endometrial-myometrial junction. The uterine cavity was also examined in both sagittal and coronal planes for structural abnormalities such as submucous fibroids, endometrial polyps, or features indicative of adenomyosis.

Evaluation of Ovaries and Adnexa

In cases where abnormal thickening or irregularities of the endometrium were identified, a detailed assessment was performed to rule out premalignant or malignant conditions. For suspected endometrial carcinoma, particular attention was paid to the extent of possible myometrial invasion, given its prognostic significance in staging and management. Following uterine assessment, the probe was angled laterally to visualize both ovaries. Each ovary was evaluated for its size, shape, and internal echotexture, with documentation of any cysts, solid masses, or atypical findings. The adnexal regions and the wider pelvic cavity were also carefully examined to exclude additional pathologies that could contribute to postmenopausal bleeding or other pelvic symptoms.

Interpretation of findings

The sonographic findings were categorized systematically for interpretation and clinical correlation. Normal endometrium was defined by appropriate thickness defined by the Royal College of Obstetricians and Gynaecologists (RCOG) as <4 mm and absence of structural irregularities [14]. Cases with thickened endometrium or abnormal architecture were flagged as suggestive of hyperplasia or other underlying pathology. Endometrial polyps were identified as well-defined, localized echogenic growths, while pyometra was characterized by an accumulation of purulent material within the uterine cavity. Features consistent with endometrial carcinoma included irregular thickening, heterogeneous echotexture, disruption of the endometrial-myometrial interface, and evidence of invasion. Submucous fibroids were recognized as hypoechoic lesions distorting the endometrial cavity. Each of these sonographic impressions was correlated with clinical presentation, and where warranted, further diagnostic confirmation through histopathological evaluation was recommended.

Ethical considerations

Ethical clearance for the study was obtained from the Institutional Ethics Committee of Sree Balaji Medical College and Hospital (002/SBMCH/IHEC/2023/2038). All participants were provided with detailed information about the study, including its purpose, procedure, benefits, and potential risks. Written informed consent was secured from each participant before enrollment, ensuring adherence to ethical principles of autonomy, confidentiality, and voluntary participation.

Statistical analysis

All collected data were entered into Microsoft Excel (Microsoft, Redmond, WA) and analyzed using SPSS version 26.0 (IBM Corp., Armonk, NY, USA). Descriptive statistics such as mean, standard deviation, frequencies, and percentages were used to summarize demographic and clinical characteristics. The association between categorical variables, such as vitamin D categories and gestational diabetes occurrence, was assessed using the chi-square test or Fisher’s exact test when appropriate. Continuous variables, such as endometrial thickness, were compared using the independent sample t-test or analysis of variance (ANOVA), depending on the number of groups. Correlation between vitamin D levels and clinical or biochemical parameters was examined using Pearson’s correlation coefficient (r). The receiver operating characteristic (ROC) curve analysis was performed to evaluate diagnostic accuracy, with the area under the curve (AUC), sensitivity, specificity, and optimal cut-off values calculated. A p-value <0.05 was considered statistically significant.

## Results

The study population consisted predominantly of postmenopausal women between 60 and 64 years of age, accounting for nearly one-third of participants, followed by 28% in the 55- to 59-year group, 20% in the 65- to 69-year group, 12% in the 50- to 54-year group, and 8% aged 70 years or above. In terms of menopausal duration, 35% had attained menopause five to nine years earlier, 30% had been menopausal for 10-14 years, 20% within the last five years, and 15% for 15 years or more. Regarding metabolic risk factors, 25% of the participants were obese (BMI≥30), while the remaining 75% were non-obese. Diabetes mellitus was present in 22% of women, whereas 78% were non-diabetic, and hypertension was reported in 30% compared to 70% who were normotensive. Reproductive history showed that 16% of the women were nulliparous, while the majority, 84%, were multiparous (Table 1).

**Table 1:** Demographic classification of the study participants (n=100)

Variable	Category/Value	Number of Participants	Percentage (%)
Age Group (years)	50-54	12	12
	55-59	28	28
	60-64	32	32
	65-69	20	20
	≥70	8	8
Duration Since Menopause	≤5 years	20	20
	5-9 years	35	35
	10-14 years	30	30
	≥15 years	15	15
Obesity Status	Obese (BMI ≥30)	25	25
	Non-obese (BMI <30)	75	75
Diabetes Mellitus	Diabetic	22	22
	Non-diabetic	78	78
Hypertension	Hypertensive	30	30
	Normotensive	70	70
Parity Status	Nulliparous	16	16
	Multiparous	84	84

In the study cohort, endometrial thickness measured ≤4 mm in 45% of the participants, while 35% had values between 5 and 8 mm, and 20% showed thickness greater than 8 mm. Assessment of serum estradiol levels revealed that half of the women had low levels (<20 pg/mL), 35% were within the normal range (20-40 pg/mL), and 15% demonstrated elevated levels (>40 pg/mL) (Table 2).

**Table 2:** Distribution of endometrial thickness and serum estradiol levels among the study participants (n =100)

Parameter	Category/Range	Number of Participants	Percentage (%)
Endometrial Thickness	≤4 mm	45	45
	5-8 mm	35	35
	>8 mm	20	20
Serum Estradiol Level	<20 pg/mL (low)	50	50
	20–40 pg/mL (normal)	35	35
	>40 pg/mL (high)	15	15

**Figure 1:**
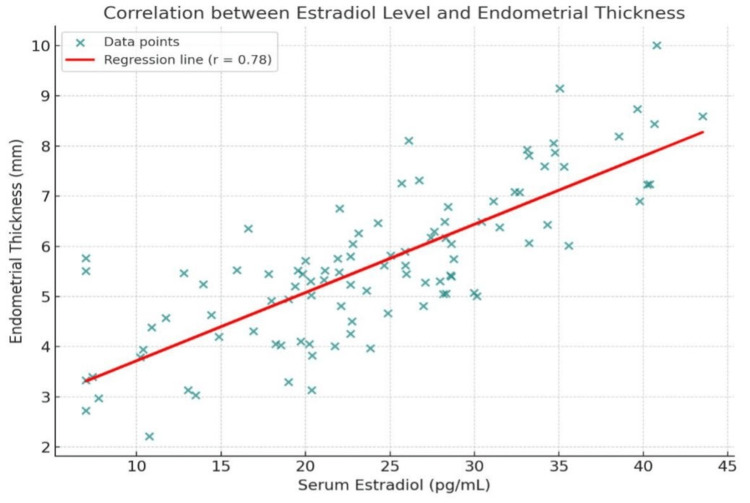
Correlation between estradiol and endometrial thickness This figure demonstrates a scatter plot showing correlation between estradiol and endometrial thickness

The correlation analysis demonstrated a statistically significant relationship between serum estradiol levels and endometrial thickness. A correlation coefficient of r=0.78 with a p-value <0.001 indicated a moderate to strong positive correlation. This finding suggests that higher estradiol levels were consistently associated with increased endometrial thickness. The strong significance reinforces the biological link between hormonal status and endometrial changes in postmenopausal women (Figure 1).

In this study of 100 postmenopausal women, ET >4 mm was found in 55 participants. The association of ET with selected risk factors is summarized in Table 3. Among participants with obesity (BMI >30, n=25), 19 (76%) had ET >4 mm and six (24%) had ET ≤4 mm, compared with 36 (48%) and 39 (52%), respectively, in participants without obesity, with a significant association (χ²=4.86, p=0.027). As regards diabetes mellitus (n=22), 17 (77%) had ET >4 mm and five (23%) had ET ≤4 mm, versus 38 (49%) and 40 (51%) in non-diabetic participants, also showing a significant association (χ²=4.56, p=0.033). Among participants with hypertension (n=30), 18 (60%) had ET >4 mm and 12 (40%) had ET ≤4 mm, compared with 37 (53%) and 33 (47%) in participants without hypertension, which was not statistically significant (χ²=0.19, p=0.661). Finally, in nulliparous participants (n=16), 13 (81%) had ET >4 mm and three (19%) had ET ≤4 mm, compared with 42 (50%) and 42 (50%) in parous participants, showing a significant association (χ²=4.12, p=0.042).

**Table 3:** Association of endometrial thickness (ET) with risk factors in the study population (n=100) Chi-square/Fischer exact test. *A p-value <0.05 is statistically significant.

Risk Factor	Total with Risk Factor (n)	ET >4 mm (n, %)	ET ≤4 mm (n, %)	Non-Risk Factor ET >5 mm (n, %)	Non-Risk Factor ET ≤5 mm (n, %)	Chi-square	p-value
Obesity (BMI>30)	25	19 (76%)	6 (24%)	36 (48%)	39 (52%)	4.86	0.027
Diabetes Mellitus	22	17 (77%)	5 (23%)	38 (49%)	40 (51%)	4.56	0.033
Hypertension	30	18 (60%)	12 (40%)	37 (53%)	33 (47%)	0.19	0.661
Nulliparity	16	13 (81%)	3 (19%)	42 (50%)	42 (50%)	4.12	0.042

## Discussion

This prospective observational study sought to elucidate the relationship between serum estradiol levels and ET in postmenopausal women, while also investigating the association of ET with key metabolic and reproductive factors. Our principal finding is a statistically significant, moderate to strong positive correlation (r=0.78, p<0.001) between serum estradiol and ET. This robust correlation substantiates the central hypothesis that the postmenopausal endometrium remains a sensitive end-organ, whose thickness, as measured by TVUS, serves as a reliable non-invasive biomarker of systemic estrogenic activity. Furthermore, our analysis revealed that obesity, diabetes mellitus, and nulliparity were significantly associated with a higher prevalence of endometrial thickening (ET >4 mm), whereas hypertension showed no significant association.

The strong positive correlation we observed is physiologically plausible and aligns with the well-established understanding of endometrial dynamics. In the absence of progesterone, estrogen acts as a potent mitogen on endometrial tissue, stimulating cellular proliferation and glandular growth. Our findings confirm that even in the postmenopausal state, this fundamental relationship persists. The distribution of our cohort - where 50% had low estradiol (<20 pg/mL) and 45% had an atrophic endometrium (≤4 mm) - reflects the expected norm of a hypoestrogenic, atrophic state for the majority. Conversely, the 15% of women with elevated estradiol levels (>40 pg/mL) were disproportionately represented in the group with ET >8 mm.

This result is strongly concordant with a large body of literature. The pivotal work of Smith-Bindman et al. established that a thin endometrial stripe (≤4 mm) on TVUS has a negative predictive value for endometrial cancer of over 99% in women with postmenopausal bleeding, effectively ruling out significant pathology in the context of low estrogen [9]. Our study extends this principle to an asymptomatic or screening population, demonstrating that ET measurement can stratify women based on their endogenous estrogen exposure. Similarly, a study by Langer et al. found that women receiving hormone therapy (HT) had significantly greater ET than non-users, and that ET measurements correlated with the dose and type of estrogen used, directly linking systemic estrogen levels to endometrial response [15]. Our findings demonstrate a significant correlation between endogenous estrogen levels and ET, providing a quantitative basis for considering ET as a surrogate marker of hormonal status in postmenopausal women, without implying that increased ET necessarily reflects elevated estrogen or pathological changes.

The finding that 76% of obese women (BMI ≥30) had an ET >4 mm, compared to only 48% of non-obese women (χ²=4.86, p=0.027), is mechanistically explained by the process of peripheral aromatization. Adipose tissue expresses the enzyme aromatase, which converts adrenal androgens (primarily androstenedione) into estrone, the primary circulating estrogen after menopause [16]. Therefore, higher adipose tissue mass directly leads to increased endogenous estrogen production, providing unopposed stimulation to the endometrium. This creates a well-documented risk profile for endometrial hyperplasia and carcinoma. Our results are consistent with those of the Nurses' Health Study and other large cohorts, which have consistently identified obesity as one of the strongest risk factors for endometrial cancer [17]. The similar association observed with diabetes (77% of diabetic women had ET >4 mm, χ²=4.56, p=0.033) is often intertwined with obesity, as insulin resistance and hyperinsulinemia can further promote endometrial proliferation through both direct mitogenic effects and by influencing sex hormone-binding globulin (SHBG) levels, thereby increasing bioavailable estrogen [18]. The co-occurrence of these metabolic factors creates a synergistic environment conducive to endometrial thickening.

Our data revealed a striking association, with 81% of nulliparous women having an ET >4 mm compared to 50% of parous women (χ²=4.12, p=0.042). This finding can be interpreted through two complementary lenses. First, nulliparity may be a marker of underlying infertility or anovulatory cycles during reproductive life, implying a history of prolonged exposure to unopposed estrogen. This chronic exposure could potentially lead to a baseline increased sensitivity of the endometrium to estrogenic stimuli later in life. Second, and more supported by evidence, is that nulliparity is an independent risk factor for endometrial cancer, as established in numerous epidemiological studies [19]. The protective effect of parity is thought to be mediated by the terminal differentiation of endometrial cells during pregnancy and the prolonged periods of high progesterone exposure, which may have a long-lasting protective effect against future estrogen-driven proliferation [20]. Our finding of thickened endometrium in nulliparous women aligns with this established risk profile, suggesting that TVUS may identify a subgroup of postmenopausal women with an inherently higher-risk endometrial environment.

The lack of a significant association between hypertension and ET (χ²=0.19, p=0.661) is an important negative finding. While hypertension is often part of the metabolic syndrome alongside obesity and diabetes, its link to endometrial pathology is likely indirect, mediated through its association with these other factors. Hypertension itself is not a direct source of estrogenic stimulation. This result suggests that when assessing endometrial risk, the focus should remain more directly on factors influencing estrogen exposure (obesity, diabetes) rather than on all components of the metabolic syndrome. However, our findings must be contextualized with studies that show limitations of using ET alone. Our study demonstrates a significant correlation between endogenous estrogen levels and endometrial thickness in postmenopausal women, supporting the use of endometrial thickness as a non-invasive marker of hormonal status. However, it is important to note that this correlation applies only to women with endogenous estrogen. Women receiving hormone replacement therapy (HRT) or medications such as tamoxifen, which exert estrogenic effects on the endometrium, often exhibit sonographically thickened, cystic, or heterogeneous endometria that may not correspond directly with serum estrogen levels [21]. 

A few limitations should be considered when interpreting these findings. The study's cross-sectional design limits the ability to establish causality between ET and estradiol levels. Additionally, the sample size may not be sufficiently large to detect subtle associations or to generalize findings to the broader population. The reliance on transvaginal ultrasound measurements, while standard, is operator-dependent and may introduce variability. Furthermore, the study did not account for all potential confounders, such as HRT use or genetic predispositions, which could influence ET measurements.

Despite these limitations, the study provides valuable insights into the relationship between ET and estradiol levels in postmenopausal women with bleeding. The findings suggest that ET measurements can serve as a useful initial screening tool for endometrial cancer, particularly when combined with other clinical assessments. However, due to the potential for false-positives, especially in women with risk factors like obesity and diabetes, further diagnostic evaluations are essential. Future research with larger, longitudinal cohorts and standardized measurement protocols is needed to validate these findings and refine screening strategies for endometrial cancer in postmenopausal women.

## Conclusions

This study demonstrates that endometrial thickness measured by transvaginal ultrasound is a reliable non-invasive biomarker of systemic estrogen levels in postmenopausal women. A strong positive correlation between serum estradiol and ET confirms that the postmenopausal endometrium remains hormonally responsive. Additionally, metabolic and reproductive factors - particularly obesity, diabetes mellitus, and nulliparity - are significantly associated with increased ET, highlighting subgroups at higher risk for endometrial proliferation and potential malignancy. Hypertension, in contrast, showed no significant effect. These findings support the clinical utility of ET measurement for risk stratification and initial screening in postmenopausal women with bleeding, emphasizing the need for individualized assessment based on hormonal and metabolic profiles. Incorporating ET evaluation into routine postmenopausal care may aid early identification of women at increased risk for endometrial pathology, guiding timely and targeted diagnostic interventions.
